# Which trace elements are accumulated in fronds of the *Athyrium filix-femina* fern? a study from the Czech Republic

**DOI:** 10.1007/s10661-025-14201-4

**Published:** 2025-06-24

**Authors:** Ivan Suchara, Václav Procházka, Julie Sucharová, Marie Holá

**Affiliations:** 1 Landscape Research Institute, Květnové náměstí 391, Průhonice, 252 43 Czech Republic; 2 Faculty of Nuclear Sciences and Physical Engineering, Czech Technical University in Prague, Břehová 7, Prague 1, 11519 Czech Republic

**Keywords:** *Athyrium filix-femina*, Element accumulation, Bioconcentration factors, Enrichment factors, Lanthanides (REE)

## Abstract

A screening test using XRF spectroscopy was done to map element concentrations in fronds of the *Athyrium filix-femina* fern growing at 244 coniferous forest plots across the Czech Republic. In the next step, 45 elements in fronds of the ferns coming from selected sites with contrasting geological and climatic conditions were determined using prevalently ICP-OES and ICP-MS methods. To our knowledge, such a number of elements analyzed is unprecedented in literature data of this important fern species. Element contents of forest floor humus, topsoil and subsoil from the same sites were adopted from previously performed analyses. To estimate element uptake and accumulation by ferns, the element distribution in fern fronds was correlated with that of soil covers and with selected site-specific factors. Bioconcentration and enrichment factors referred to the element contents in forest floor humus and soil were used for estimation of fern abilities to accumulate elements in fronds. Besides accumulation of macronutrients, fern fronds moderately accumulated Ba, Cd, Hg, Mo, Mn, Ni, Zn and mainly lanthanides (REEs). Only several of the trace elements concentrated in granites (Rb, Cs, Be, Tl, U) have significant positive correlation between contents in fronds and those in mineral soil. Anthropogenic pollution was mainly relevant for Fe in the Ostrava industrial region.

**Supplementary information:**

The online version contains supplementary material available at 10.1007/s10661-025-14201-4.

## Introduction

Some of the about 12,000 fern species, frequently from the Pteridaceae family, accumulate or hyperaccumulate some trace elements, including several elements of major toxicological interest like As, in sporophytes and gametophytes (e.g. Eslava-Silva et al., 2023; Meharg, 2003; Srivastava et al., 2010; Xie et al., 2009). This is also true for several of about 180 species of the *Athyrium* genus (Van et al., 2006; Zhang et al., 2014). Element accumulation in fern bodies frequently increases in the following order: fronds < rhizomes < roots. Across fern fronds, petioles usually had lower element concentrations than blades (e.g. Jedynak et al., 2009; Samecka-Cymerman et al., 2011).

*Athyrium filix-femina* is widespread across three continents (Meusel et al., 1965), and several intraspecies taxa and cultivars have been recognized and cultivated. *Athyrium filix-femina* (further *AFF*) occurs across the whole territory of the Czech Republic except for the warmest regions of lowlands (Kaplan et al., 2018); however, there are no available data on its elemental composition there.

There are several studies on elemental composition of *AFF* from Poland, including the Czech-Polish border regions. Samecka-Cymerman et al. (2011) found relatively low concentrations of Cr, Al, Fe and V in *AFF* in the Giant Mts. Nevertheless, some authors reported accumulation of Ni (Parzych & Jonczak, 2018a, 2018b) and As (e.g., Jedynak et al., 2009). Also, the ability of *AFF* (similar to many other ferns) to accumulate lanthanides was documented (Grosjean et al., 2020).

The basis of our study is the determination of contents of 46 elements in fronds of *AFF* in the Czech territory at various geological units. The composition of green biomass of various other plants (moss, grass, spruce) from the sites sampled was evaluated by Suchara et al., (2011, 2017). These plants are valuable indicators of pollution for many elements; however, no significant accumulation of trace elements from the substrate has been found in the species investigated. Here, we concentrate on the influence of element contents in substrates (humus and two mineral soil horizons) and other selected site explanatory factors on element contents in fronds, with some emphasis on the ability of *AFF* to accumulate trace elements. Besides, in the “Discussion”, we summarize available literature data on the elemental composition of *AFF* fronds in Europe (except for studies with experimental chemical treatment) to facilitate the comparison of our large dataset (which is relatively unique for a single fern species) with data from other countries.

## Material and methods

### Samples

Specimens of *AFF* were collected at 244 forest plots of ca 50 × 50 m included in a network of the national moss biomonitoring campaigns (Suchara et al., 2017). Two middle-aged fronds, frequently with developed spore sacks, growing between the inner and outermost fronds were collected from 3 to 5 different fern plants within each sampling plot in August and September 2005. A photo of AFF clumps is included in Supplementary Materials. The leaves from each plot were joined into a composite sample. The samples were air dried, milled and sieved for particle diameters ≤ 0.25 mm. The dried leaves weighed 50–130 g, corresponding to about 300–600 g of fresh leaves (Prats et al., 2024).

### Element content determination

**Fig. 1:**
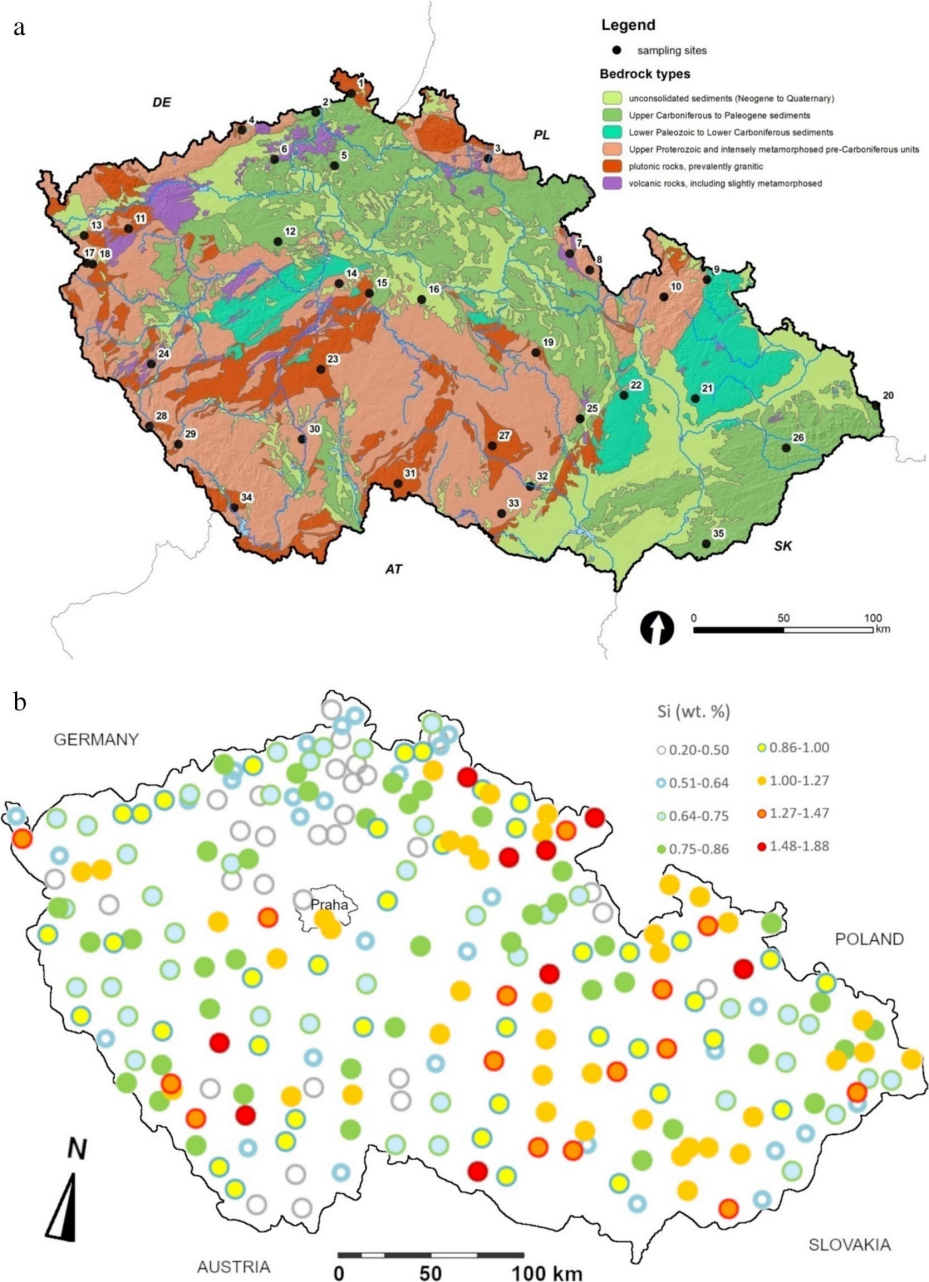

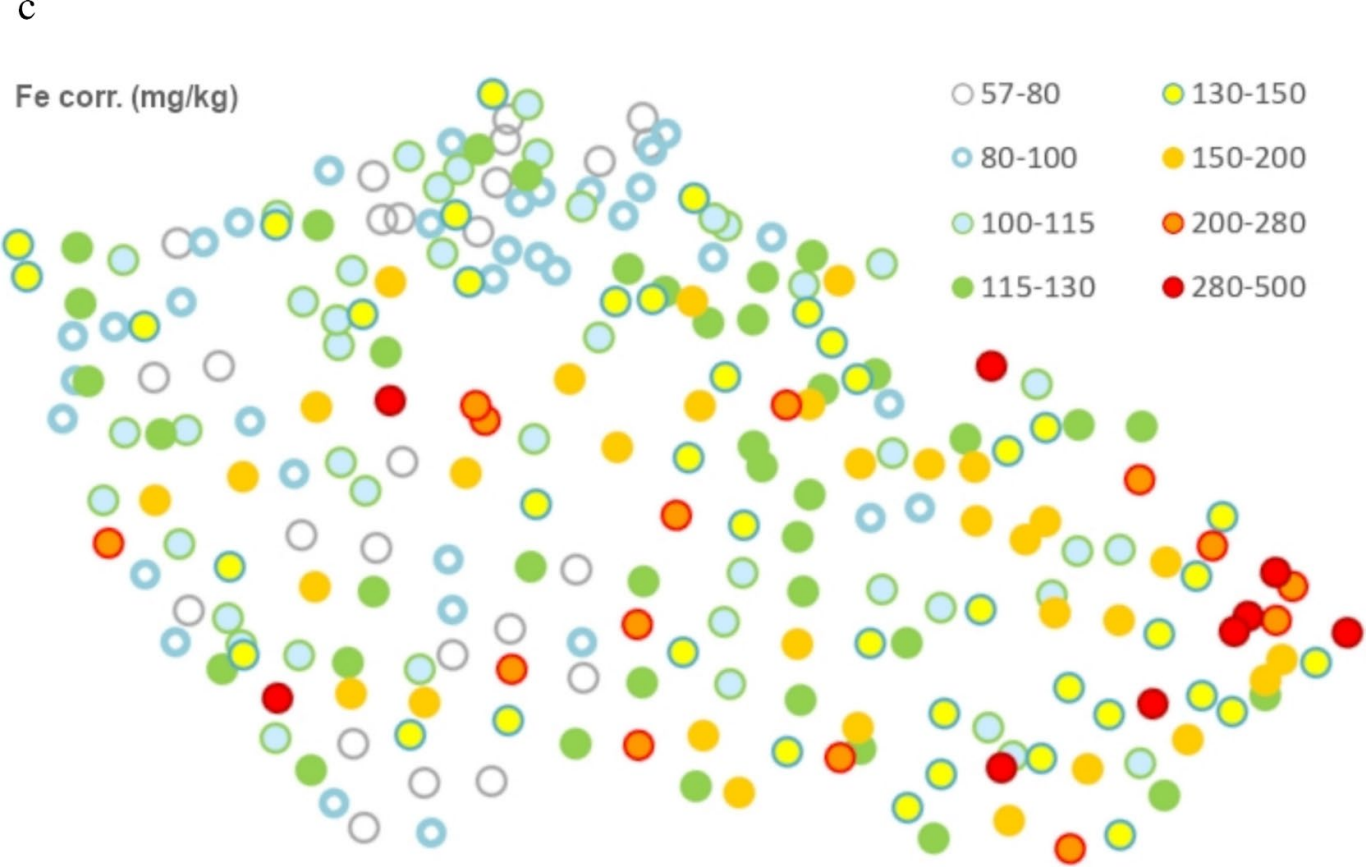
**a** Distribution of selected 35 sampling plots of *AFF* in a simplified geological map of the Czech Republic. **b** Map of Si content in fronds from all sites, determined by XRF. **c** Map of Fe contents determined by XRF and corrected by a simple linear function according to Fe determination by OES in 35 overlapping samples

A screening of elements heavier than Na in the powdered fern samples stored in ziplock bags was performed using a portable XRF analyser VANTA VRM (Olympus). The analytical mode “Geochemistry”, a spot diameter 10 mm, a sequence of two maximum beam energies 40 and 10 kV and automatic interpretation of spectra were used. The ziplock bags (made of polyethylene 0.05 mm thick) with samples were placed immediately at the window protecting the X-ray source and detector. The results of the XRF measurements (including other fern- and diverse plant species) will be published separately; however, the Si- and partly Fe contents obtained are utilized in this study. For more detailed elemental analyses, 35 frond specimens coming from sites of contrasting geological and climatic characteristics were selected (Table 1, Fig. 1).

**Table 1:** Geographical coordinates, elevation, bedrock (with simplified classification into 5 groups—see ESM 3) and position in climatic areas and potential annual evaporation of the *AFF* sampling plots

Sampling plot order	Latitude °N	Altitude °E	Elevation m a. s. l	Bedrock*	Bedrock classification	Climatic zone**	Potential evaporation (mm/y)**
1	50.9399	14.4360	460	Quartzose sandstone	SAND	MW7	550–600
2	50.8210	14.1769	410	Quartzose, argillaceous and green sandstone	SAND	MW7	550–600
3	50.7045	15.5760	790	Phyllite	PHYL	C6	500–550
4	50.6795	13.6199	770	Granite	GRA	C5	500–550
5	50.5688	14.3818	305	Quartzose sandstone	SAND	W2	600–650
6	50.5586	13.9075	570	Trachyte, altered basalt	BAS	W2	600–650
7	50.2787	16.3015	635	Greywacke and phyllite	PHYL	C6	500–550
8	50.2075	16.4717	715	Gneiss	PAR	C6	500–550
9	50.2206	17.3922	835	Muscovitic-chloritic and chloritic schist with phyllite	PHYL	MW1	550–600
10	50.1126	17.0680	855	Biotite and two-mica gneiss to augen schist	PAR	MW1	500–550
11	50.1053	12.8571	520	Granites and granodiorite	GRA	MW6	550–600
12	50.1518	14.0255	420	Sandy marlite, spiculite and claystone	SAND	MW11	650–700
13	50.0350	12.5249	555	Granite	GRA	W2	600–650
14	49.9861	14.5441	320	Greywacke	PHYL	MW11	650–700
15	49.9586	14.7875	485	Granite	GRA	MW7	600–650
16	49.9614	15.1957	365	Paragneiss	PAR	W2	650–700
17	49.9004	12.5784	585	Paragneiss to migmatite	PAR	MW3	550–600
18	49.9008	12.6253	600	Paragneiss	PAR	MW6	550–600
19	49.7649	16.1267	700	Paragneiss, gneiss to migmatite	PAR	W2	650–700
20	49.6649	18.7873	610	Sandstone and claystone in the Carpathian flysch	SAND	MW1	550–600
21	49.6206	17.3902	325	Clay shale, siltstone and greywacke	PHYL	MW7	600–650
22	49.6005	16.8391	585	Greywackes and clayey shale	PHYL	W2	650–700
23	49.5472	14.4917	635	Porphyry amphibole-biotite granite (durbachite)	GRA	MW4	550–600
24	49.4509	13.1954	535	Andesitic basalt	BAS	MW11	600–650
25	49.4599	16.5224	480	Two-mica paragneiss with garnets	PAR	MW4	550–600
26	49.4164	18.1225	565	Sandstone and claystone in the Carpathian flysch	SAND	C7	550–600
27	49.2755	15.8751	555	Granite to quartz syenite (durbachite)	GRA	MW4	550–600
28	49.1419	13.2536	925	Paragneiss	PAR	C6	550–600
29	49.0740	13.4929	910	Granite	GRA	C6	500–550
30	49.1878	14.4242	440	Paragneiss	PAR	MW7	550–600
31	49.0307	15.1937	685	Granite	GRA	MW2	550–600
32	49.0953	16.2002	275	Serpentine, peridotite	BAS	MW11	650–700
33	48.9429	16.0065	355	Migmatite to orthogneiss	PAR	MW7	650–700
34	48.7999	13.9835	880	Granite, melanocratic granite, granodiorite, migmatite	GRA	MW1	550–600
35	48.9003	17.5799	405	Sandstone and marlite in the Carpathian flysch	SAND	W2	650–700

*Adopted from ČGS (2024)

**Adopted from Tolasz et al. (2007)

*C* cold, *MW* moderately warm, *W* warm climatic areas

The pulverized sample was dried (40 °C/4 h), and then, 0.25 g was weighed in a Teflon digestion vessel. After adding 6 ml HNO_3_ and 2 ml H_2_O_2_, the sample was subtotally digested in a CEM MARS 6 microwave digestion system (1200 W/200 °C/20 min). The humus and soil samples (0.200 g) were digested in a similar way, but a two-step procedure was used: 1. HNO_3_ + H_2_O_2_ + HF = 5.0: 2.0: 0.3–0.6 ml, 2. H_3_BO_3_ = 3–6 ml according to preliminarily determined Si contents.

Contents of the following elements were determined by solution analyses: Al, B, Ba, Ca, Fe, K, Mg, Mn, Na, P, S, Sr, Zn and (in soil and humus only) Si by ICP-OES (Avio 500, Perkin Elmer) and Ag, As, Be, Bi, Cd, Ce, Co, Cr, Cs, Cu, Ga, Ge, La, Li, Mo, Nd, Ni, Pb, Pr, Rb, Sb, Se, Sn, Th, Tl, U, V, W and Y by ICP-MS (NexION 2000, Perkin Elmer). Total Hg and C and N contents were measured directly in powdered samples using a single-purpose mercury AMA 256 atomic absorption spectrometer and a carbon and nitrogen determinator (LECO CN 928), respectively.

All measurements were performed in three independent replications (see ESM 1 for complete data). Blind samples and reference materials (IAEA Lichen 336, NIST Pine Needles 1575a, NIST Apple Leaves and LECO ALPHALPHA 502–237) were analyzed in parallel (see ESM 2 for results of reference materials analyses). No recalibration of analytical instruments had to be done.

### Explanatory factors

The analytical results were related to the selected sampling plots specific characteristics, such as elevation, bedrock types and potential annual evaporation. More explanatory factors, namely pH values and element contents of humus, topsoil and subsoil of the sampling sites, were adopted from the previous measurements (Suchara et al., 2011).

### Indices of element accumulation

To estimate the efficiency of *AFF* to accumulate elements in fronds, the bioconcentration factor (BcF) was used (e.g. Burnison, 1998):

1 $$\text{BcF }=\text{ cEf}/\text{cEs}$$

where cE_f_ and cE_s_ represent the content of E element in frond and in soil, respectively.

Enrichment factors (EFs) (Barbieri, 2016) were computed as ratios of normalized element content in *AFF* to normalized element content in humus (O_h_), topsoil and subsoil, respectively, using Al for chemical normalization:

2 $$(\text{EF}) = (\text{E}/\text{Al})\text{frond}/(\text{E}/\text{Al})\text{substrate}$$

Not enriched, slightly, moderately, severely, highly severely and extremely enriched classes were defined by the respective intervals < 1.5, [1.5;2], [2;5], [5;20], [20;40] and ≥ 40.

### Cerium anomalies calculation

Cerium anomalies (Ce/Ce*) were calculated using geometric extrapolation to obtain the theoretical Ce* value (i.e. Ce concentration in case of no anomaly) by the following formula:

3 $$Ce/Ce* = CeN/[PrN2/NdN]$$

where Ce_N_, Pr_N_ and Nd_N_ are element values, normalized to the element contents in Post-Archean Australian Shales (see Barrat et al., 2023 for details). Values of Ce/Ce* significantly lower and greater than 1 mean a negative and a positive anomaly, respectively.

### Statistics

**Fig. 2:**
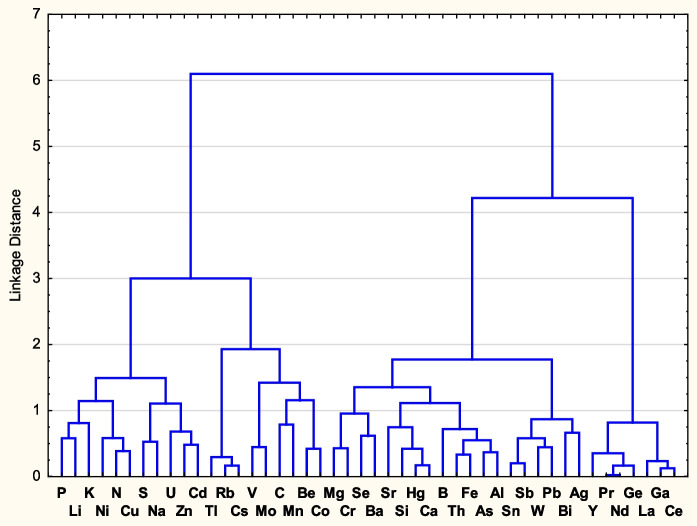
Cluster analysis (Ward’s method, 1-Pearson *r*, Distance Linkage) dendrogram for the medians of element contents in AFF at 35 sampling sites. Medians used were log_10_ transformed

**Fig. 3:**
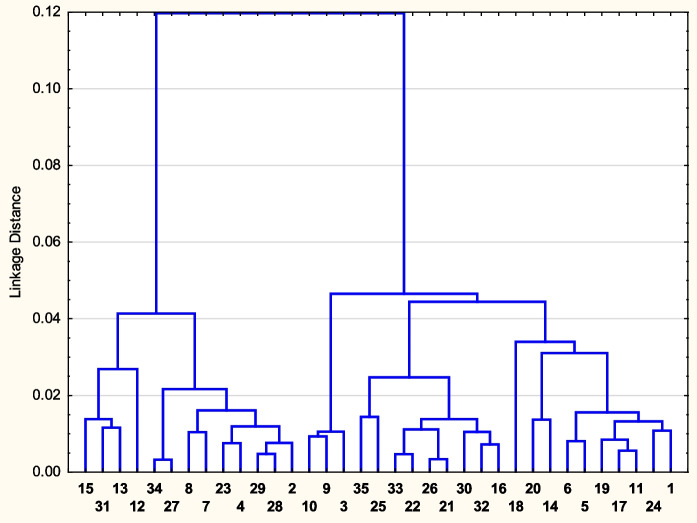
Cluster analysis (Ward’s method, 1-Pearson *r*, Distance Linkage) dendrogram for 35 sampling sites of AFF with the given medians of element contents. Medians used were log_10_ transformed

Basic statistics and statistical tests and analyses were obtained in the StatSoft STATISTICA 14.0 software. Measured variables having a typically log-normal distribution were log transformed (log_10_ median) for statistical evaluations. Due to remaining differences from normal distribution in some transformed data, Spearman’s correlation coefficients were computed in all correlation analyses used. For cluster analysis in STATISTICA, the following parameters were used: raw variable or cases data (for Figs. 2 and 3, respectively), Ward’s method and 1–Pearson r for cluster, amalgamation rule and distance measure, respectively.

## Results

The element contents and their basic statistics for the *AFF* fronds specimens are presented in Table 2.

**Table 2:** Basic statistics for the element contents in the *AFF* fronds (mg/kg), *n* = 35, *SD* standard deviation, *RSD* relative standard deviation, *I.Q.* first quartile, *III.Q.* third quartile, *MAD* median absolute deviation

Element	Min		Max	Mean	SD	RSD (%)	I.Q	Median	III.Q	MAD
C	428,968		465,741	449,293	8952	2	445,000	449,229	454,000	5064
N	20,223		36,254	25,926	3643	14	23,050	25,889	28,100	2615
Ag	0.008		0.103	0.017	0.016	91	0.011	0.014	0.018	0.004
Al	50.4		260	138	56.1	40	96.7	129	170	40.4
As	0.024		0.242	0.084	0.047	54	0.050	0.072	0.100	0.024
B	16.3		77.0	27.8	9.78	35	23.2	25.4	30.9	2.67
Ba	53.1		306	136	59.0	43	94.1	127	168	38.7
Be	0.004		0.302	0.043	0.064	147	0.012	0.018	0.049	0.010
Bi	0.001		0.025	0.004	0.004	95	0.002	0.003	0.006	0.001
Ca	4260		10,763	6730	1701	25	5398	6248	8044	986
Cd	0.044		1.63	0.339	0.319	93	0.132	0.581	0.411	0.159
Ce	1.53		48.9	21.6	10.6	49	14.2	19.1	28.8	5.85
Co	0.036		0.833	0.171	0.153	90	0.082	0.114	0.222	0.045
Cr	0.307		1.85	0.489	0.261	53	0.375	0.421	0.509	0.075
Cs	0.016		20.0	1.88	3.68	193	0.179	0.415	2.47	0.319
Cu	5.04		15.3	9.24	2.22	23	7.92	9.02	10.0	1.12
Fe	72.0		300	127	44.3	34	102	112	142	14.8
Ga	0.084		0.421	0.225	0.083	36	0.167	0.208	0.251	0.043
Ge	0.086		0.488	0.220	0.091	41	0.162	0.208	0.243	0.041
Hg	0.021		0.110	0.045	0.025	54	0.027	0.036	0.054	0.010
K	14,455		32,592	23,264	4723	20	19,827	23,762	27,044	3390
La	1.62		72.4	18.4	13.0	70	11.6	15.6	20.8	4.58
Li	0.077		7.25	0.899	1.62	167	0.153	0.425	0.652	0.271
Mg	2023	6516		3318	1011	31	2752	3010	3804	425
Mn	34.1	931		146	157	112	88.35	104	142.0	22.8
Mo	0.098	1.65		0.388	0.356	96	0.166	0.234	0.443	0.104
Na	5.28	266		46.7	60.4	136	12.40	21.0	52.70	11.1
Nd	1.75	35.4		10.9	7.64	62	6.56	8.22	12.65	2.43
Ni	0.744	26.9		5.32	7.40	132	1.61	2.63	5.56	1.42
P	1102	3740		2012	643	31	1518	1913	2318	406
Pb	0.171	2.78		0.684	0.599	72	0.312	0.415	0.733	0.165
Pr	0.388	8.51		3.00	1.87	58	1.97	2.46	3.35	0.581
Rb	7.54	517		138	138	104	49.10	78.0	200	31.9
S	1339	2528		1832	264	15	1655	1852	1978	159
Sb	0.009	0.062		0.023	0.013	46	0.015	0.019	0.029	0.006
Se	0.34	0.498		0.190	0.115	58	0.104	0.159	0.249	0.061
Sn	0.029	0.122		0.056	0.024	38	0.041	0.046	0.068	0.010
Sr	9.67	64.2		28.7	14.3	50	16.70	25.4	34.15	9.18
Th	0.003	0.039		0.086	0.107	51	0.009	0.044	0.024	0.026
Tl	0.004	0.588		0.017	0.009	130	0.026	0.015	0.124	0.007
U	0.002	0.681		0.032	0.115	383	0.005	0.007	0.014	0.004
V	0.190	1.67		0.572	0.356	64	0.368	0.427	0.559	0.104
W	0.003	0.027		0.011	0.006	54	0.008	0.010	0.013	0.002
Y	0.207	35.1		3.23	5.95	70	0.988	1.57	2.975	0.953
Zn	19.7	56.7		28.0	8.10	30	23.15	26.4	28.95	3.27
Si	2973	13,344		8386	2623	31	6312	8749	10,116	2022

Results of cluster analysis for the elements in 35 fern samples and for the 35 selected sampling sites are shown in Figs. 2 and 3.

Correlation coefficients for element contents in *AFF* with element contents in the substrates and the explanation factors are gathered in ESM 4. Bioconcentration factors for the element contents in *AFF* related to the element contents in humus, the topsoil and subsoil are shown in ESM 5 and enrichment factors in ESM 6.

### Macroelements

Of elements analyzed, macroelements include C, N, P, K, Ca, Mg, S, Fe and Si. They play structural, nutritional and protective roles in plant bodies. Contents of C (median 449.2 ± 5.06 g/kg) showed extremely low variability (CV 1.1%), and the sampling site factors did not significantly affect C content in *AAF* fronds. All significant correlations between the content of C and of other elements are negative (except for the positive correlation with Cr).

Nitrogen contents (20.2–36.3 g/kg) significantly positively correlated with P, S and Cu contents and negatively with Ba in fronds. The C:N ratios ranged from 11.9 to 22.6 and N:P ratios from 5.4 to 21.6.

Contents of P in fronds showed a wide range (1.10–3.74 g/kg). Fronds from sites with granitic bedrocks had significantly higher P concentrations and lower C:P and N:P ratios.

The relatively high contents of K in fronds (14.4–32.6 g/kg) are consistent with other studies (Tables 2 and 3). The K contents positively and negatively correlated with granitic and phyllitic bedrocks, respectively. Potassium negatively correlated with Ca and Mg in fronds and with humus pH.

**Table 3:** Published data of element contents and bioconcentration factors in *AFF* fronds (mg/kg, unless stated otherwise) determined in European natural or seminatural biotopes

Element	Range	Mean (median)	BcF	Habitats	Decomposition + analytical methods	Source
Al	10–200*	110	0.01–0.02*^+^	Alder forest riverbank marsh, N Poland	HNO_3_+H_2_O_2_,MP-AES	Parzych & Jonczak, 2018b
	103–484	354	0.04^m^	Forests in the Kaczawskie Mts., SW Poland	HNO_3_+HClO_4_, ETAAS	Samecka-Cymerman et al., 2011
As	2.2–2.8		n.d.	Metalliferous soils, N Italy	HNO_3_, ICP-OES	Cornara et al., 2007
		20*	0.01*^m^	Soil in Ag-As mining area, S Poland	HNO_3_, GFAAS	Jedynak et al., 2009
C (%)		42		Peat birch forest, Baltic shore, N Poland	K_2_Cr_2_O_7_, titration Mohr's	Parzych, 2010
Ca		3350		Wet spruce forest, NE Poland	burn, FAES	Czerwiński & Pracz, 1995
	1000–7000*	4000*	0.38–0.52*^+^	Alder forest riverbank marsh, N Poland	HNO_3_+H_2_O_2_, FAAS	Parzych & Jonczak, 2018b
	4907–6879		100–220*	Headwater riparian alder forest, N Poland	HNO_3_+H_2_O_2_, FAAS	Parzych & Astel, 2018
	5464–9900	7232	10^+m^	Forests in the Kaczawskie Mts., SW Poland	HNO_3_+HClO_4_, FAAS	Samecka-Cymerman et al., 2011
	1120–6300*	2140*		Polluted and unpolluted sites in Norway and Lithuania	n.s., AAS	Stapulionytė et al., 2018
	3400–11100			Coniferous forests on soddy podzols, Moscow region	n.s.	Vtorova & Solntseva 1983
Cd	0.11–1.25	0.139		Metalliferous soils, N Italy	HNO_3_, ICP-OES	Cornara et al., 2007
	0.061–0.387	(0.087)	0.13^m^	Eastern Giant Mts., S Poland	HNO_3_+H_2_O_2_, GFAAS	Krawczyk et al., 2006
	0.10–0.91	0.38 (0.35)	0.9^+m^	Forest soils, mining and smelting area, NE Slovakia	Aqua Regia, GFAAS	Musilova et al., 2016
		0.27	0.55^•m^	Beech woods on acid and damp soils, Germany	GFAAS/ICP-OES	Neite et al., 1991
	0.01–0.10	0.06		Forests in the Kaczawskie Mts., SW Poland	HNO3+HClO_4_, ETAAS	Samecka-Cymerman et al., 2011
	0.013–1.30*	0.08*		Polluted and unpolluted sites in Norway and Lithuania	n.s., AAS	Stapulionytė et al., 2018
Co	0.004–0.144	0.065 (0.074)	0.08^+m^	Eastern Giant Mts., S Poland	HNO_3_+H_2_O_2_, GFAAS	Krawczyk et al., 2006
	0.1–0.6	0.3	0.04^+m^	Forests in the Kaczawskie Mts., SW Poland	HNO3+HClO_4_, ETAAS	Samecka-Cymerman et al., 2011
Cr	14.6–44.7		n.d.–0.01	Metalliferous soils, N Italy	HNO_3_, ICP-OES	Cornara et al., 2007
	7.39–11.8	8.95 (8.62)	3.0^+m^	Eastern Giant Mts., S Poland	HNO_3_+H_2_O_2_, GFAAS	Krawczyk et al., 2006
	0.1–0.4	0.2	0.01^+m^	Forests in the Kaczawskie Mts., SW Poland	HNO3+HClO_4_, ETAAS	Samecka-Cymerman et al., 2011
Cu	6.2–18.2		0.31–0.36	Metalliferous soils, N Italy	HNO_3_, ICP-OES	Cornara et al., 2007
	9.15–11.9	10.7 (10.9)	0.94^m^	Eastern Giant e Mts., S Poland	HNO_3_+H_2_O_2_, FAAS	Krawczyk et al., 2006
	3.34–85.7	10.3 (7.61)	0.09^+m^	Forest soils, mining and smelting area, NE Slovakia	Aqua Regia, FAAS	Musilova et al., 2016
		19	24^m^	Beech woods on acid and damp soils, Germany	GFAAS/ICP-OES	Neite et al., 1991
	n.d.–29*	12.5*	1.2–1.7*^+^	Alder forest riverbank marsh, N Poland	HNO_3_+H_2_O_2_, FAAS	Parzych & Jonczak, 2018b
	7.2–16*	9.7*	0.4*^+m^	Forests in the Kaczawskie Mts., SW Poland	HNO3+HClO_4_, ETAAS	Samecka-Cymerman et al., 2011
	1.4–18*	6.3*		Polluted and unpolluted sites in Norway and Lithuania	n.s., AAS	Stapulionytė et al., 2018
Fe	130–198		n.d.–0.01	Metalliferous soils, N Italy	HNO_3_, ICP-OES	Cornara et al., 2007
	41–255	116 (109)	0.03^+m^	Eastern Giant Mts., S Poland	HNO_3_+H_2_O_2_, FAAS	Krawczyk et al., 2006
	90–310*	200*	0.02–0.03*^+^	Alder forest river bank marsh, N Poland	HNO_3_+H_2_O_2_, FAAS	Parzych & Jonczak, 2018b
	99–415	216	0.01^+m^	Forests in the Kaczawskie Mts., SW Poland	HNO3+HClO_4_, FAAS	Samecka-Cymerman et al., 2011
	22–1070*	115*		Polluted and unpolluted sites in Norway and Lithuania	n.s., AAS	Stapulionytė et al., 2018
Hg	n.d.–6.59	0.22 (0.05)	n.d.–1.23	Forest soils, polluted by Cu and Hg smelters, NE Slovakia	AAS	Árvay et al., 2017
	0.02–12.8	0.99 (0.06)	0.03^m^	Forest soils, mining and Cu and Hg smelters, NE Slovakia	Hg-AAS (AMA-254)	Musilova et al., 2016
K (%)		0.757		Wet spruce forest, NE Poland	ashing, FAES	Czerwiński & Pracz, 1995
	2.1–3.2*	2.63*	14.7–39.7*^+^	Alder forest riverbank marsh, N Poland	HNO_3_+H_2_O_2_, FAAS	Parzych & Jonczak, 2018b
	1.69–2.19		4760–20335*^w^	Headwater riparian alder forest, N Poland	HNO_3_+H_2_O_2_, FAAS	Parzych & Astel, 2018
	1.44–2.86	2.02	37^+m^	Forests in the Kaczawskie Mts., SW Poland	HNO3+HClO_4_, FAAS	Samecka-Cymerman et al., 2011
	1.23–2.51*	1.48*		Polluted and unpolluted sites in Norway and Lithuania	n-a., AAS	Stapulionytė et al., 2018
	3.83–5.02			Coniferous forests on soddy podzols, Moscow region	n.s.	Vtorova & Solntseva, 1983
Mg		5180		Wet spruce forest, NE Poland	FAAS	Czerwiński & Pracz, 1995
	2500–4600*	3500*	1.7–3.9*^+^	Alder forest riverbank marsh, N Poland	HNO_3_+H_2_O_2_, FAAS	Parzych & Jonczak, 2018b
	3781–4068		590–3270*^w^	Headwater riparian alder forest, N Poland	HNO3+HClO_4_, FAA	Parzych & Astel, 2018
	2172–5137	3994	1.6^+m^	Forests in the Kaczawskie Mts., SW Poland	HNO3+HClO_4_, FAAS	Samecka-Cymerman et al., 2011
	1230–10000*	1900*	0.63^+m^	Polluted and unpolluted sites in Norway and Lithuania	n.s., AAS	Stapulionytė et al., 2018
	2800–6700			Spruce and pine forest on soddy podzols, Moscow region	n.s.	Vtorova & Solntseva, 1983
Mn	53–141	101 (107)	1.0^+m^	Eastern Giant Mts., S Poland	HNO_3_+H_2_O_2_, FAAS	Krawczyk et al., 2006
	45–130*	86.5*	0.25–0.35*^+^	Alder forest riverbank marsh, N Poland	HNO_3_+H_2_O_2_, FAAS	Parzych & Jonczak, 2018b
	194–519	339		Forests in the Kaczawskie Mts., SW Poland	HNO3+HClO_4_, FAAS	Samecka-Cymerman et al., 2011
	10–500*	45*		Polluted and unpolluted sites in Norway and Lithuania	n.s., AAS	Stapulionytė et al., 2018
N (%)		2.380		Wet spruce forest, NE Poland	Kjeldahl	Czerwiński & Pracz. 1995
		1.711	1.7^m^	Peat birch forest, Baltic shore, N Poland	Kjeldahl	Parzych, 2010
	1.06–1.83*	1.413*		Headwater riparian alder forest, N Poland	Kjeldahl	Parzych & Jonczak, 2018a
	1.00–2.36*	1.676*	1.5–1.8*^+^	Alder forest riverbank marsh, N Poland	Kjeldahl	Parzych & Jonczak, 2018b
	1.40–1.41		1.62–5.42*^w^	Headwater riparian alder forest, N Poland	Kjeldahl	Parzych & Astel, 2018
	2.69–2.94			Coniferous forests on soddy podzols, Moscow region	n.s.	Vtorova & Solntseva, 1983
Na		650		Wet spruce forest, NE Poland	ashing, FAES	Czerwiński & Pracz, 1995
	4–500*	40*		Polluted and unpolluted sites in Norway and Lithuania	n.s., AAS	Stapulionytė et al., 2018
	500–700			Coniferous forests on soddy podzols, Moscow region	n.s.	Vtorova & Solntseva, 1983
Ni	25.8–33.9		0.06–0.15	Metalliferous soils, N Italy	HNO_3_, ICP-OES	Cornara et al., 2007
	4.49–8.54	6.65 (6.65)	2.8^+m^	Eastern Giant Mts., S Poland	HNO_3_+H_2_O_2_, GFAAS	Krawczyk et al., 2006
	13–42*	26*	2.8–3.9*^+^	Alder forest riverbank marsh, N Poland	HNO_3_+H_2_O_2_, FAAS	Parzych & Jonczak 2018b
	1.1–4.9	2.4	0.18^+m^	Forests in the Kaczawskie Mts., SW Poland	HNO3+HClO_4_, ETAAS	Samecka-Cymerman et al., 2011
P	468–2379			Metalliferous soils, N Italy	HNO_3_, ICP-OES	Cornara et al., 2007
		1720		Wet spruce forest, NE Poland	Spectrophotometry	Czerwiński & Pracz, 1995
		4000		Boreal forests	n.s.	Gerloff et. al., 1964 ex Larsen, 1982
		1410	1.3^m^	Peat birch forest, Baltic shore, N Poland	Spectrophotometry	Parzych, 2010
	1500–3450*	2485*	0.36–0,40*^+^	Alder forest riverbank marsh, N Poland	Spectrophotometry	Parzych & Jonczak, 2018b
	2177–2266		5385–29230*^w^	Headwater riparian alder forest, N Poland	Spectrophotometry	Parzych & Astel, 2018
	108–1367	858	2.9^+m^	Forests in the Kaczawskie Mts., SW Poland	Spectrophotometry	Samecka-Cymerman et al., 2011
	3400–4600			Coniferous forests on soddy podzols, Moscow region	n.s.	Vtorova & Solntseva, 1983
Pb	0.90–1.34		n.d.–0.03	Metalliferous soils, N Italy	HNO_3_, ICP-OES	Cornara et al., 2007
	2.84–13.1	5.34 (4.45)	0.05^+m^	Eastern Giant Mts., S Poland	HNO_3_+H_2_O_2_, GFAAS	Krawczyk et al., 2006
	0.09–12.3	2.21 (1.10)	0.04 ^+m^	Forest soils, mining and smelting area, NE Slovakia	Aqua Regia, GFAAS	Musilova et al., 2016
		24	0.6^•m^	Beech woods on acid and damp soils, Germany	GFAAS/ICP-OES	Neite et al., 1991
	2.6–9.2	5.3	0.14^+m^	Forests in the Kaczawskie Mts., SW Poland	HNO3+HClO_4_, ETAAS	Samecka-Cymerman et al., 2011
	0.07–99*	0.08*		Polluted and unpolluted sites in Norway and Lithuania	GFAAS/ICP-OES	Stapulionytė et al., 2018
					HNO3+HClO_4_, ETAAS	
S	700–1400	1230		Wet pine forest, NE Poland	n.s.	Czerwiński & Pracz, 1995
				Coniferous forests on soddy podzols, Moscow region	n.s.	Vtorova & Solntseva, 1983
Si (%)	0.63–1.25			Coniferous forests on soddy podzols, Moscow region	n.s.	Vtorova & Solntseva, 1983
Sr	29–61*	44*	0.76–0.99*^+^	Alder forest riverbank marsh, N Poland	HNO_3_+H_2_O_2_,MP-AES	Parzych & Jonczak, 2018b
V	0.2–2.4	0.9	0.04^+m^	Forests in the Kaczawskie Mts., SW Poland	HNO3+HClO_4_, ETAAS	Samecka-Cymerman et al., 2011
Zn	14.6–44.7		0.32–0.58	Metalliferous soils, N Italy	HNO_3_, ICP-OES	Cornara et al., 2007
	21.7–50.5	30.1 (26.5)	0.76^+m^	Eastern Giant Mts., S Poland	HNO_3_+H_2_O_2_, FAAS	Krawczyk et al., 2006
	12.4–73.7	39.5 (38.3)	0.21^+m^	Forest soils, mining and smelting area, NE Slovakia	Aqua Regia, FAAS	Musilova et al., 2016
		71	3.4^•m^	Beech woods on acid and damp soils, Germany	GFAAS/ICP-OES	Neite et al., 1991
	12–32*	22.5*	0.36–0.55*^+^	Alder forest riverbank marsh, N Poland	HNO_3_+H_2_O_2_, FAAS	Parzych & Jonczak, 2018b
	40–91	61	1.0^+m^	Forests in the Kaczawskie Mts., SW Poland	HNO3+HClO_4_, FAAS	Samecka-Cymerman et al., 2011
	10–102*	15*		Polluted and unpolluted sites in Norway and Lithuania	n.s., AAS	Stapulionytė et al., 2018

*Estimated from published diagrams, ^+^ values related to subtotal element contents, ^w^ BcF related to the element contents in surface water, ^*^ BcF related to the extractable (NH_4_Cl) element contents in soil, *m* mean value, *n.a.* not available

The fronds had twofold higher contents of Ca than Mg, which is generally consistent with the literature. Although both sites with peak Mg contents in fronds (6.5 and 5.95 g/kg) have Mg-rich rock substrates (serpentinite and basalt, respectively), there is no statistically significant correlation of Mg or Ca with their contents in soil. Frond Ca content correlates positively with humus pH.

Sulphur content in fronds (1.3–2.5 g/kg) correlated positively (besides N) with Na, Zn and Cu and with phyllitic bedrocks. Interestingly, there is no correlation of S in fronds and in humus, but a strong negative correlation of S in fronds and in the upper mineral horizon B1 (in deeper mineral horizon B2, S was not analyzed).

Iron content in fronds is, on average, similar to that of Mn, and it is positively correlated with Ca, Mg, Si and many trace elements like Cr. A map of Fe contents obtained by XRF is shown in Fig. 1c, where a regional enrichment in the very east (near Ostrava) is documented.

The XRF measurements showed relatively high median Si contents in *AFF* fronds: 8.12 ± 1.85 and 8.75 ± 1.56 g/kg from the whole country (*n* = 244) and from the studied plots (*n* = 35), respectively. Contents of Si showed a strong positive correlation with Ca, Fe, Al and many trace elements. The spatial distribution of Si contents is shown in Fig. 1b.

### Microelements

Concentrations of B, Cu and Zn in fronds showed the lowest variability among the trace elements, indicating a physiological control to maintain optimal content, while the highest variability of U, Cs, Li, Tl, Be, Ni and even Na may imply low *AFF* control on uptake and accumulation of these elements. Average content of Mn is strongly influenced by an outlying peak value 931 mg/kg, while the range of other samples is 34–381 mg/kg.

Increased content of some microelements was associated with certain bedrock types, namely granites and durbachite (Be, Rb, Cs, Tl), sandstones (Zn) and phyllites (Pb, W; influence of the sulfidic mineralization associated with gold deposits near site 9 is possible). The site 32 with chemically contrasting serpentinite bedrock also has a high content of Cr, Ni and Cu in fronds, but the whole group of basic and ultrabasic rocks is not distinct.

Positive correlations prevail among microelement contents in fronds. Very strong mutual positive correlations showed REEs and Y, which also positively correlated with Ga, Ge, Al and Si. Contents of Cs, Rb, Sr and Tl in fronds correlated positively, and Cr, Cu, Li and Ni correlated negatively with altitude. Potential evaporation, negatively correlating with elevation (*r* = –0.65**), positively affected Co, Li and Th contents and negatively affected Bi, Ca, Tl and Rb contents.

### Cerium anomalies

The fronds prevalently showed negative cerium anomalies with Ce/Ce* ratios 0.33–1.38, mean 0.85, SD 0.26, median 0.84 and MAD 0.21. The only three fern samples with significant positive Ce anomalies are those with the highest Mn concentrations.

### Bioconcentration factors

The BcF values (see ESM 5 for complete data) mostly decreased when related to the element contents in the following order: humus > topsoil > subsoil; however, frequently, the highest BcF values were found when related to the topsoil (ESM 5). The average BcF > 1 showed some macroelements (Ca, K and Mg) and microelements (Ba, Rb, Ce, La, Nd, Pr and Y) when BcF was related to the humus element contents, while when compared with element contents in the lower mineral soil, the BcF exceeded 1 for K, Mg, Cd, Cu, Hg, Mn, Rb and Zn. Despite relatively high BcFs for Rb, the K/Rb ratios in fronds were generally higher than in humus and soil. Probably, a high BcF for B would have also been indicated; however, B content was not determined in substrates. On the other hand, Na, As, Bi, Pb, Sb, Sn, Th, Tl and W showed medians BcFs < 0.1 related to all soil layers, indicating that these elements are excluded by *AFF*. BcFs were relatively significantly influenced by site factors (see “Discussion” and ESM 7).

### Enrichment factors

Normalized element contents in the subsoil, topsoil and humus were used as local background values for computing enrichment factors of elements in fronds. Due to relatively low Al content and enrichment in Cd, Hg, Bi, Cu, Mo, Pb, Sb and Sn (partly due to atmospheric deposition) in humus, the EFs medians except for REE and alkali elements are higher when related to element contents in soil (mainly in the subsoil) than to that in humus. Elements with no to slight enrichment in fronds were Ag, As, Bi, Cr, Fe, Li, Na, Sb, Sn, Th, U, V and W, mainly if related to the humus element contents. The highest median EFs characterize macronutrients and their analogues (Ba, Ca, Cs, K, Mg, Rb, S, Sr), some micronutrients and toxic elements (Cd, Cu, Hg, Mn, Mo, Zn) and especially REE (La, Ce, Pr, Nd) and Y.

## Discussion

### Cluster analysis

Figure 2 shows clusters of elements, whose variability in *AFF* is similar partly due to their generally similar chemical properties and common occurrence (e.g. lanthanides, or Rb and Cs). Biogenic elements tend to concentrate in the left cluster. Figure 3 shows clusters of sampling sites with most similar medians of element contents in AFF. The left cluster (13 sites) contains almost all (8 of 9) sampling sites with granitic bedrock. However, other bedrock groups are little distinct, most likely due to their internal variability and the importance of the remaining explaining variables, such as the elevation, potential evaporation or climate (Table 1).

### Macroelements

Regarding carbon, the only relevant publication found (Parzych, 2010) stated a lower average C content in fronds of *AFF* (420 g/kg) growing in wet soils of coniferous forests in comparison to our data from drier forest soils (Table 3). The negative correlations between concentrations of C and of many other elements are due to a dilution of element pools during the growth of the frond biomass.

Nitrogen contents significantly positively correlated with other nutrients—P and S. The C:N ratios (11.9–22.6) were not significantly affected by the site elevation, potential evaporation and soil pH. Literature data (Table 3) are at disposal mainly from Poland, where mostly lower N content has been reported (associated with higher C:N ratios, e.g. about 24.5 in the fronds of *AFF* from muddy habitats studied by Parzych, 2010).

Contents of P in fronds showed greater variability than N and a wider range than in the most of literature data (Table 3). Fronds from sites with granitic bedrocks had significantly higher P concentrations and lower C:P and N:P ratios due to commonly increased P content in granites. The N:P ratios reflect an instantaneous fern nutritional status and local element uptake limitations (Güsewell, 2004; Koerselman & Meuleman, 1996). The N and P limitations for *AFF* growth were indicated for 17 and 9 sampling plots, respectively.

The positive correlation of K in fronds with granitic bedrock rather reflects the content of K-feldspar than the total K in the rocks, because liberation of K from some minerals like the white mica can be very slow. Negative correlation with Ca and Mg in fronds and with humus pH may also reflect the influence of bedrocks.

While in our data, fronds from only one site had higher content of Mg than Ca, Czerwiński and Pracz (1995) found higher content of Mg than Ca (5.18 vs. 3.34 g/kg) in fronds of *AFF* growing in peat soil (i.e. in a probably Mg-poor environment).

Sulphur content in fronds was higher than in the two studies from literature available (Table 3).

Iron contents in fronds are comparable to literature data. Iron is the only element documented to be regionally enriched in the major eastern industrial region between the city Ostrava and the Polish and Slovak borders (see also Fig. 1c). In contrast, the strong pollution in the northwestern region (part of the “Black triangle”) which peaked in the 1980 s has already not unequivocally influenced the distribution of any of the elements analyzed in ferns at the time of collection (2005), although it was still slightly visible in the distribution of elements in moss (Suchara et al., 2017). Similarly, the role of industrial pollution on Fe content in AFF was documented in comparison of various regions in Lithuania and Norway (Stapulionytė et al., 2018).

Contents of Si, playing structural and protective roles in plants, showed a strong positive correlation with humus pH and with many elements in fronds, e.g. Ca, some of which (e.g. Al, Ga, Th, lanthanides, partly Fe) are typical for mineral dust (as also manifested in their spatial distribution in moss—see Suchara et al., 2011, 2017). However, if we consider Al as the representative of mineral (wind-transported) dust, the ratios of other elements to Al can be used to estimate the maximum dust content (see also enrichment factors calculated from ratios of other elements to Al in ESM 6). It follows that the dust may only little contribute to the pools of elements like Si or REEs in the fronds. The Si content in fronds is low mainly on the most quartz-rich sandstones in the northern part of the Bohemian Cretaceous Basin. This can be explained by the fact that Si is mainly liberated by weathering of silicates (like feldspars) and only insignificantly from quartz.

### Microelements

Obtained contents of microelements in fronds (Table 2) are comparable with available published data. Some microelements (e.g. B, Cu, Mn, Mo and Zn) in low concentrations serve as essential micronutrients while in higher concentrations they are toxic.

Concentrations of B, Cu and Zn in fronds showed the lowest variability among the trace elements, indicating a physiological control to maintain optimal content, in contrast to highly variable Cs, Li, Th and Be. The most contrasting bedrock type influencing directly trace elements in biomass is granitic rocks (Rb, Cs, Be, Tl, U). Some other rocks may be chemically very contrasting as well (e.g. quartz-rich sandstones, ultrabasic rocks), but the group of sandstones is probably highly heterogeneous (e.g. some of them can be relatively Ca-rich, or intercalated with other sediments), and basic and carbonate substrates are relatively rare in Czech coniferous forests. The trace elements concentrated in granites (Rb, Cs, Be, Tl, U) are the only elements with significant correlation between contents in fronds and those in mineral soil. In literature, an example of bedrock-dominated variability of some elements in AFF fronds was presented by Samecka-Cymerman et al. (2011) from the Giant Mts.

The peak uranium content in fronds at the site 18 (where U is also high in humus but not in mineral soil) in a former U mining area may reflect a contamination by U-enriched dust. Some role of atmospheric deposition and/or historic pollution can be also expected (in addition to gas-forming elements—N, S, Se) for elements whose contents in fronds are significantly correlated to those in humus: Mn, Sb, Sn and Zn. On the other hand, Se in fronds correlates negatively with Se in humus, and S and Zn in fronds correlate negatively with S and Zn, respectively, in mineral soil. This could indicate significant mobility of these elements in the plant-soil system.

Positive correlations like Fe–Ni and Cd-Cu–Zn in fronds may reflect associations of these elements in minerals in both bedrock and soil. Some positive correlations of elements in fronds (e.g. Hg with Al or Fe) which are missing in humus and soil could be explained by other factors, like pH. Significant negative correlations were found seldom (except for those of other elements, e.g. Ba, Cr, Ga, Se, Si and W with C), e.g. Fe-Rb, Na-Pb, K-Pb, K-Hg and Mn-Cd. Negative (with borderline statistical significance, however) correlation of both Ba and Pb with P in fronds may be caused by their decreased bioavailability in phosphorus-rich soils, as documented for Pb (Miretzky & Fernandez-Cirelli, 2008). Humus pH correlates positively with B, Ca, Ce, Ge, Hg, Mg, Th and Si and negatively with Cd, K and Rb. Contents of Rb, Si and Th in fronds correlated positively, and Li and Ni correlated negatively with elevation. Potential evaporation, negatively correlating with elevation (*r* = –0.70; *p* < 10^−3^), positively affected Co, Li, Na and Th contents and negatively affected Bi, Cs, Rb and Tl contents.

However, some significant correlations may be accidental due to the coincidence of many factors (e.g. elevation correlates negatively with evaporation and positively with granitic rocks).

### Cerium anomalies

The prevalently negative cerium anomalies and the fact that only the three Mn-richest fern samples have significant positive Ce anomalies imply that the fern prevalently takes up REE from solutions. Soil solutions (similar to surface water) tend to be depleted in Ce^3+^ due to its oxidation to little soluble Ce^4+^ which can be bound to Fe- and Mn oxides and hydroxides (Bau & Koschinsky, 2009). No significant relationships of Ce anomalies to the site factors were found.

### Bioconcentration factors

In contrast to element contents alone, the bioconcentration factors were frequently significantly influenced by site factors (see also ESM 7). For example, humus pH significantly negatively affected BcF for Ba, Be, Ca, Cd, Co, Cs, K, Mg, Mn, Na, Rb, Sr and Zn, while the elevation showed mainly positive effects on BcF, significantly for Ca, Cs, La, Nd, Pr, Rb, Si, Sr, Tl and Y. Some significant correlations of BcF related to the element contents in humus were found for bedrock types, e.g. negative correlations with sandstones (Cd, Cs, Cu, Ga, Ge and Tl), and positive with granites (Cs, Rb, Tl and U).

The site factors, except for humus pH, influence the BcFs related to mineral soil more (but partly differently) than BcFs related to humus. This is consistent with the assumption that *AFF* uptakes elements mainly from deeper horizon than humus. Note that in case of two sandstone sites (Nos. 2 and 5), high BcFs simply reflect extremely low contents of most elements in the mineral soil. However, such an explanation cannot be applied to elevated BcFs for elements concentrated in granites at sites with granitic bedrock.

Published BcFs determined in several studies (except for values related to water or mud in wetlands) (Table 3) did not show hyperaccumulation of any element by *AFF*. BcFs slightly exceeding the value 1.00 for (potentially) toxic elements Cr, Cu, Mn, Ni and Zn were reported seldom. Accumulation of these elements in *AFF* is explained by the effects of some bedrock types and frequently by increased atmospheric deposition rates of heavy metals at sites affected by industrial emissions (Krawczyk et al., 2006). Our study has not confirmed considerable accumulation of heavy metals in *AFF* in the country.

### Enrichment factors

Due to relatively low Al content and enrichment in Cd, Hg, Bi, Cu, Mo, Pb, Sb and Sn (partly due to atmospheric deposition) in humus, the EFs medians except for REE and alkali elements are higher when related to element contents in soil (mainly in the subsoil) than to that in humus. Elements with no to slight enrichment in fronds were Ag, As, Bi, Cr, Fe, Li, Na, Sb, Sn, Th, U, V and W, mainly if related to the humus element contents. The highest median EFs characterize macronutrients and their analogues (Ba, Ca, Cs, K, Mg, Rb, S, Sr), some micronutrients and toxic elements (Cd, Cu, Hg, Mn, Mo, Zn) and especially REE (La, Ce, Pr, Nd) and Y. Reasons for bioaccumulation of REE in *AFF* fronds are not clear; however, recent studies admit some beneficial roles of REE in plants (e.g. Kovaříková et al., 2019, and references therein).

Considerable inter-plots EFs variability includes severe enrichment of Ni, Co, Sn and Tl at few sites, and severe enrichments of Cd, Cu, Hg, Mo and Zn at many sites. pH in humus and partly in the topsoil negatively correlated with EFs for many elements (mainly Ca, Cd, Mg) when related to elements in various soil horizons. Significant influence of other site factors on EFs related to humus, topsoil or subsoil was limited to few elements. Potential evaporation had negative effects on all EFs of Rb. Sandstone bedrocks positively affected EFs (related to subsoil) for Fe, Mg, Mn, Na, Ni and Zn, while EFs of Cs, Rb, Tl and U (related to humus) positively correlated with granite bedrocks.

## Conclusions

Median EF values showed bioaccumulation of Ca, K, Mg, Mn, Ba, Rb, Y and REEs in fronds of *AFF* populations across the country. Not considering macronutrients, the greatest median EF values (ca. 100–500) (in relation to humus and normalized by Al) were obtained for Rb, La, Pr, Nd and Ce, while Cu, Zn, Mn, Mo, Ba, Sr, Cd, Cs, Tl and Y have median EFs between 10 and 100. Significant accumulation of the remaining microelements was not indicated (EFs < 10). REEs are accumulated in *AFF* fronds very strongly in comparison to other little soluble elements like Al; however, from the viewpoint of bioconcentration factors (all median BCFs < 10), *AFF* cannot be denoted as a hyperaccumulator of any of the trace elements analyzed. Our results indicate that *AFF* is rather an excluder of Na, Al, Fe and many trace elements (Ag, As, Bi, Cr, Li, Sb, Sn, Th, U, V and W).

## Supplementary information

Below is the link to the electronic supplementary material.

Supplementary file1 (XLSX 41.8 KB)

Supplementary file2 (DOCX 20 KB)

Supplementary file3 (XLSX 105 KB)

Supplementary file4 (XLSX 42 KB)

Supplementary file5 (XLSX 96 KB)

Supplementary file6 (XLSX 94 KB)

Supplementary file7 (DOCX 47 KB)

Supplementary file8 (JPG 2720 KB)

## Data Availability

No datasets were generated or analysed during the current study.
