# Supplementary material for: Which trace elements are accumulated in fronds of the Athyrium filix-femina fern? a study from the Czech Republic
Source: Environ Monit Assess. 2025 Jun 24;197(7):801. doi: 10.1007/s10661-025-14201-4 (PMC12187792; doi:10.1007/s10661-025-14201-4)
Supplement: Supplementary file 2 — Supplementary file2 (DOCX 20 KB) [file 10661_2025_14201_MOESM2_ESM.docx]

Table s1 Certified and informative element contents (mg/kg ± 95% confidence interval) in the standard reference materials used and determined element contents in the laboratory (n.s. = not stated)

| **IAEA Lichen 336** | | | **NIST Pine Needles 1575a** | | |
| --- | --- | --- | --- | --- | --- |
| **Element** | **Certified value**  ***Informative value** | **Determined**  **(n = 12)** | **Element** | **Certified value**  ***Informative value** | **Determined**  **(n = 18)** |
| **(Ag)** | **n.s.** | (0.021±0.001) | **(Ag)** | **n.s.** | (0.016±0.002) |
| **Al*** | **680±110*** | 661±8.58 | **Al** | **580±30** | 565±14 |
| **As** | **0.63±0,08** | 0.628±0.018 | **As** | **0.039±0.002** | 0.038±0.002 |
| **Ba** | **6.40±1.10** | 6.37±0.045 | **Ba** | **6.0±0.2** | 5.66±0.067 |
| **(Be)** | **n.s.** | (0.020±0.0017) | **Be** | **n.s.** | (<0.005) |
| **(Bi)** | **n.s.** | (0.017±0.0062) | **Bi** | **n.s.** | (<0.005) |
| **Ca*** | **2800±450*** | 2675±39.2 | **Ca** | **2500±100** | 2465±47.4 |
| **Cd*** | **0.117±0.017*** | 0.103±0.002 | **Cd** | **0.233±0.004** | 0.210±0.009 |
| **Ce** | **1.28±0.17** | 1.15±0.15 | **Ce*** | **0.11*** | 0.10 |
| **Co** | **0.29±0.05** | 0.298±0.006 | **Co** | **0.061±0.002** | 0.060±0.003 |
| **Cr*** | **1.06±0.17*** | 1.05±0.04 | **Cr*** | **0.3 – 0.5*** | 0.508±0.016 |
| **Cs** | **0.11±0.013** | 0.104±0.001 | **Cs** | **0.283±0.009** | 0.276±0.003 |
| **Cu** | **3.6±0.5** | 3.53±0.07 | **Cu** | **2.8±0.2** | 2.85±0.157 |
| **Fe** | **430±50** | 412±5 | **Fe** | **46±2** | 44.4±0.399 |
| **(Ga)** | **n.s.** | (0.197±0.007) | **Ga** | **n.s.** | (0.036±0.001) |
| **(Ge)** | **n.s.** | (0.064±0.01) | **Ge** | **n.s.** | (0.014±0.001) |
| **K** | **1840±200** | 1759±40 | **K** | **4170±70** | 4012±61 |
| **La** | **0.66±0.10** | 0.614±0.016 | **La*** | **0.053±0.008** | 0.047±0.001 |
| **(Li)** | **n.s.** | (0.447±0.011) | **Li** | **n.s.** | (0.120±0.005) |
| **Mg*** | **580±110*** | 530±3.118 | **Mg** | **1060±170** | 977±17 |
| **Mn** | **63±7** | 60.6±0.66 | **Mn** | **488±12** | 481±9 |
| **(Mo)** | **n.s.** | (0.068±0.002) | **Mo** | **n.s.** | (0.018±0.001) |
| **Na** | **320±40** | 290±6 | **Na** | **63±1** | 61.3±2.9 |
| **Nd** | **0.60±0.18** | 0.515±0.015 | **Nd** | **n.s.** | (0.043±0.001) |
| **(Ni)** | **n.s.** | (0.975±0.019) | **Ni** | **1.47±0.10** | 1.42±0.05 |
| **P** | **610±120** | 587±15.0 | **P** | **1070±80** | 1006±60.2 |
| **Pb*** | **4.9±0.6*** | 4.61±0.124 | **Pb** | **0.167±0.015** | 0.158±0.006 |
| **(Pr)** | **n.s.** | (0.130±0.004) | **Pr** | **n.s.** | (0.011±0.001) |
| **Rb*** | **1.76±0.22*** | 1.60±0.08 | **Rb** | **16.5±0.9** | 15.9±0.7 |
| **S** | **n.s.** | (602±6.863) | **S** | **n.s.** | (771±22) |
| **Sb** | **0.073±0.01** | 0.064±0.002 | **Sb*** | **0.0062 – 0.033** | 0.005 |
| **Se** | **0.22±0.04** | 0.202±0.006 | **Se** | **0.099±0.004** | 0.091±0.008 |
| **(Sn)** | **n.s.** | (0.121±0.004) | **Sn** | **n.s.** | (0.034±0.001) |
| **Sr** | **9.3±1.1** | 8.97±0.07 | **Sr*** | **6.8±1.5** | 6.74±0.09 |
| **Th** | **0.14±0.02** | 0.149±0.023 | **Th*** | **0.016±0.001** | 0.022±0.005 |
| **(Tl)** | **n.s.** | (0.009±0.001) | **Tl** | **n.s.** | (0.018±0.000) |
| **(U)** | **n.s.** | (0.033±0.002)7 | **U** | **n.s.** | (0.005±0.0002) |
| **V*** | **1.47±0.22*** | 1.44±0.035 | **V** | **n.s.** | (0.117±0.003) |
| **(W)** | **n.s.** | (0.010±0.002) | **W** | **n.s.** | (0.007±0.001) |
| **(Y)** | **n.s.** | (0.349±0.010) | **Y** | **n.s.** | (0.035±0.001) |
| **Zn** | **30.4±3.4** | 28.8±1.4 | **Zn** | **38±2** | 36.9±0.5 |
|  | **NIST Apple Leaves 1515** | | **LECO ALFALFA 502-273** | | |
|  | **Certified** | **Determined**  **(n = 15)** |  | **Certified** | **Determined**  **(n = 15)** |
| **Hg** | **0.0432±00023** | 0.042±0.001 | **C** | **443000±2000** | 442720±1330 |
| **N** | **22990±900** | 22400±200 |  | | |
