# Supplementary material for: Which trace elements are accumulated in fronds of the Athyrium filix-femina fern? a study from the Czech Republic
Source: Environ Monit Assess. 2025 Jun 24;197(7):801. doi: 10.1007/s10661-025-14201-4 (PMC12187792; doi:10.1007/s10661-025-14201-4)
Supplement: Supplementary file 7 — Supplementary file7 (DOCX 47 KB) [file 10661_2025_14201_MOESM7_ESM.docx]

**Pearson correlation coefficients (statistically significant correlations only) between bioconcentration factors and site factors**

| **BcF/humus** |  | **Ba** | **Be** | **Ca** | **Cd** | **Co** | **Cs** | **Hg** | **K** | **Mg** | **Mn** | **Na** | **Rb** | **Se** | **Sr** | **Zn** | **C** |
| --- | --- | --- | --- | --- | --- | --- | --- | --- | --- | --- | --- | --- | --- | --- | --- | --- | --- |
| **pH humus** | **r** | **-0.455** | **-0.39** | **-0.808** | **-0.648** | **-0.469** | **-0.34** | **0.346** | **-0.689** | **-0.364** | **-0.708** | **-0.499** | **-0.578** | **0.430** | **-0.395** | **-0.461** | **0.603** |
|  | **p** | **0.006** | **0.021** | **0.000** | **0,000** | **0.004** | **0.046** | **0.044** | **0.000** | **0.037** | **0.000** | **0.002** | **0.000** | **0.010** | **0.010** | **0.005** | **0.000** |
|  |  |  |  |  |  |  |  |  |  |  |  |  |  |  |  |  |  |
| **Elevation** |  | **Ca** | **Cs** | **La** | **Nd** | **Pr** | **Rb** | **Si** | **Sr** | **Tl** | **Y** | **C** |  |  |  |  |  |
|  | **r** | **0.412** | **0.482** | **0.422** | **0.349** | **0.366** | **0.588** | **0.441** | **0.388** | **0.611** | **0.342** | **-0.352** |  |  |  |  |  |
|  | **p** | **0.014** | **0.003** | **0.012** | **0.04** | **0.03** | **0.000** | **0.008** | **0.021** | **0,000** | **0.044** | **0.038** |  |  |  |  |  |
|  |  |  |  |  |  |  |  |  |  |  |  |  |  |  |  |  |  |
| **Evaporation** |  | **Cs** | **Li** | **Na** | **Rb** | **Th** | **Tl** | **Y** |  |  |  |  |  |  |  |  |  |
|  | **r** | **-0.348** | **0.389** | **0.364** | **-0.672** | **-0.358** | **-0.366** |  |  |  |  |  |  |  |  |  |  |
|  | **p** | **0.041** | **0.024** | **0.370** | **0.000** | **0.035** | **0.044** |  |  |  |  |  |  |  |  |  |  |
|  |  |  |  |  |  |  |  |  |  |  |  |  |  |  |  |  |  |
|  |  |  |  |  |  |  |  |  |  |  |  |  |  |  |  |  |  |
| **Sandstones** |  | **Cd** | **Cs** | **Cu** | **Ga** | **Ge** | **Tl** |  |  |  |  |  |  |  |  |  |  |
|  | **r** | **-0.381** | **-0.396** | **-0.361** | **-0.368** | **-0.361** | **-0.467** |  |  |  |  |  |  |  |  |  |  |
|  | **p** | **0.024** | **0.019** | **0.032** | **0.030** | **0.033** | **0.005** |  |  |  |  |  |  |  |  |  |  |
|  |  |  |  |  |  |  |  |  |  |  |  |  |  |  |  |  |  |
| **Granites** |  | **Cs** | **Rb** | **Tl** | **U** |  |  |  |  |  |  |  |  |  |  |  |  |
|  | **r** | **0.518** | **0.543** | **0.524** | **0.375** |  |  |  |  |  |  |  |  |  |  |  |  |
|  | **p** | **0.000** | **0.001** | **0.001** | **0.026** |  |  |  |  |  |  |  |  |  |  |  |  |
|  |  |  |  |  |  |  |  |  |  |  |  |  |  |  |  |  |  |
| **Phyllites** |  | **Sr** |  |  |  |  |  |  |  |  |  |  |  |  |  |  |  |
|  | **r** | **0.398** |  |  |  |  |  |  |  |  |  |  |  |  |  |  |  |
|  | **p** | **0.018** |  |  |  |  |  |  |  |  |  |  |  |  |  |  |  |
|  |  |  |  |  |  |  |  |  |  |  |  |  |  |  |  |  |  |
| **Basalts** |  | **Cr** | **Mg** |  |  |  |  |  |  |  |  |  |  |  |  |  |  |
|  | **r** | **-0.414** | **-0.364** |  |  |  |  |  |  |  |  |  |  |  |  |  |  |
|  | **p** | **0.013** | **0.037** |  |  |  |  |  |  |  |  |  |  |  |  |  |  |
|  |  |  |  |  |  |  |  |  |  |  |  |  |  |  |  |  |  |
| **Gneiss** |  | **Cu** | **V** |  |  |  |  |  |  |  |  |  |  |  |  |  |  |
|  | **r** | **0.363** | **0.344** |  |  |  |  |  |  |  |  |  |  |  |  |  |  |
|  | **p** | **0.032** | **0.043** |  |  |  |  |  |  |  |  |  |  |  |  |  |  |
|  |  |  |  |  |  |  |  |  |  |  |  |  |  |  |  |  |  |
|  |  |  |  |  |  |  |  |  |  |  |  |  |  |  |  |  |  |
| **BcF/topsoil** |  | **Be** | **Ca** | **Cd** | **Co** | **Cs** | **Hg** | **Mg** | **Mn** | **Rb** | **Si** | **Sr** |  |  |  |  |  |
| **pH humus** | **r** | **-0.369** | **-0.436** | **-0.606** | **-0.568** | **-0.429** | **0.405** | **0.400** | **-0.347** | **-0.549** | **0.454** | **-0.427** |  |  |  |  |  |
|  | **p** | **0.020** | **0.009** | **0.000** | **0.000** | **0.010** | **0.016** | **0.017** | **0.041** | **0.001** | **0.006** | **0.010** |  |  |  |  |  |
|  |  |  |  |  |  |  |  |  |  |  |  |  |  |  |  |  |  |
| **Elevation** |  | **Al** | **As** | **Be** | **Bi** | **Cu** | **Fe** | **Ga** | **Hg** | **Li** | **Na** | **Ni** | **Rb** | **Se** | **Th** | **Tl** | **V** |
|  | **r** | **-0.355** | **0.400** | **-0.414** | **-0.371** | **-0.474** | **-0.551** | **-0.575** | **-0.468** | **-0.584** | **-0.358** | **-0.346** | **0.398** | **-0.536** | **-0.347** | **0.403** | **-0.371** |
|  | **p** | **0.036** | **0.007** | **0.013** | **0.038** | **0.004** | **0.001** | **0,000** | **0.005** | **0,000** | **0.005** | **0.042** | **0.018** | **0.001** | **0.041** | **0.016** | **0.028** |
|  |  |  |  |  |  |  |  |  |  |  |  |  |  |  |  |  |  |
| **Evaporation** |  | **Al** | **As** | **Be** | **Fe** | **Ga** | **Hg** | **Li** | **Na** | **Rb** | **S** | **Se** | **V** | **C** | **N** |  |  |
|  | **r** | **0.343** | **0.455** | **0.361** | **0.359** | **0.464** | **0.343** | **0.6** | **0.343** | **-0.401** | **-0.375** | **0.408** | **0.359** | **0.513** | **0.571** |  |  |
|  | **p** | **0.044** | **0.006** | **0.033** | **0.034** | **0.005** | **0.044** | **0.000** | **0.019** | **0.017** | **0.026** | **0.015** | **0.034** | **0.002** | **0.000** |  |  |
|  |  |  |  |  |  |  |  |  |  |  |  |  |  |  |  |  |  |
|  |  |  |  |  |  |  |  |  |  |  |  |  |  |  |  |  |  |
| **Sandstones** |  | **Ag** | **Al** | **As** | **Ba** | **Be** | **Bi** | **Ca** | **Cd** | **Cr** | **Fe** | **Ga** | **Hg** | **K** | **Li** | **Mg** | **Mn** |
|  | **r** | **0.453** | **0.46** | **0.396** | **0.495** | **0.382** | **0.167** | **0.559** | **0.466** | **0.389** | **0.615** | **0.339** | **0.354** | **0.615** | **0.347** | **0.474** | **0.361** |
|  | **p** | **0.006** | **0.005** | **0.019** | **0.002** | **0.024** | **0.005** | **0.000** | **0.005** | **0.021** | **0.000** | **0.046** | **0.037** | **0.000** | **0.041** | **0.004** | **0.033** |
|  |  |  |  |  |  |  |  |  |  |  |  |  |  |  |  |  |  |
| **Granites** |  | Ag | **Al** | **As** | **Bi** | **Ca** | **Ga** | **Ni** | **Pb** | **S** | **Sn** | **Th** | **W** | **Y** | **Zn** |  |  |
|  | **r** | **-0.356** | **-0.362** | **0.420** | **-0.531** | **-0.369** | **-0.395** | **0.350** | **-0.511** | **-0.427** | **-0.642** | **-0.492** | **-0.544** | **-0.369** | **-0.401** |  |  |
|  | **p** | **0.036** | **0.032** | **0.012** | **0.001** | **0.029** | **0.019** | **0.040** | **0.002** | **0.010** | **0.000** | **0.003** | **0.001** | **0.029** | **0.017** |  |  |
|  |  |  |  |  |  |  |  |  |  |  |  |  |  |  |  |  |  |
| **Phyllites** |  | **S** | **Sn** | **W** |  |  |  |  |  |  |  |  |  |  |  |  |  |
|  | **r** | **0.345** | **0.390** | **0.390** |  |  |  |  |  |  |  |  |  |  |  |  |  |
|  | **p** | **0.042** | **0.020** | **0.020** |  |  |  |  |  |  |  |  |  |  |  |  |  |
|  |  |  |  |  |  |  |  |  |  |  |  |  |  |  |  |  |  |
| **Basalts** |  | **Co** | **Mn** | **Sr** | **U** |  |  |  |  |  |  |  |  |  |  |  |  |
|  | **r** | **-0.354** | **-0.354** | **-0.354** | **-0.344** |  |  |  |  |  |  |  |  |  |  |  |  |
|  | **p** | **0.037** | **0.037** | **0.037** | **0.043** |  |  |  |  |  |  |  |  |  |  |  |  |
|  |  |  |  |  |  |  |  |  |  |  |  |  |  |  |  |  |  |
|  |  |  |  |  |  |  |  |  |  |  |  |  |  |  |  |  |  |
|  |  |  |  |  |  |  |  |  |  |  |  |  |  |  |  |  |  |
|  |  |  |  |  |  |  |  |  |  |  |  |  |  |  |  |  |  |
|  |  |  |  |  |  |  |  |  |  |  |  |  |  |  |  |  |  |
| **BcF/subsoil** |  | **Be** | **Ca** | **Cd** | **Co** | **Cs** | **Hg** | **Si** | **Sr** | **Th** |  |  |  |  |  |  |  |
| **pH humus** | **r** | **-0.368** | **-0.435** | **-0.519** | **0.501** | **-0.429** | **0.541** | **0.511** | **-0.431** | **0.423** |  |  |  |  |  |  |  |
|  | **p** | **0.030** | **0.009** | **0.001** | **0.002** | **0.010** | **0.001** | **0.002** | **0.010** | **0.011** |  |  |  |  |  |  |  |
|  |  |  |  |  |  |  |  |  |  |  |  |  |  |  |  |  |  |
| **Elevation** |  | **Al** | **As** | **Be** | **Bi** | **Cr** | **Cu** | **Fe** | **Ga** | **Hg** | **La** | **Li** | **Na** | **Ni** | **Th** | **Tl** | **V** |
|  | **r** | **-0.344** | **-0.467** | **0.383** | **-0.303** | **-0.422** | **-0.501** | **-0.542** | **-0.484** | **-0.61** | **-0.649** | **-0.357** | **-0.391** | **-0.449** | **-0.353** | **0.537** | **-0.344** |
|  | **p** | **0.043** | **0.005** | **0.024** | **0.032** | **0.012** | **0.002** | **0.001** | **0.003** | **0,000** | **0.000** | **0.035** | **0.020** | **0.007** | **0.037** | **0.035** | **0.043** |
|  |  |  |  |  |  |  |  |  |  |  |  |  |  |  |  |  |  |
| **Evaporation** |  | **As** | **Cu** | **Fe** | **Ga** | **Hg** | **Li** | **Na** | **Ni** | **Th** | **V** |  |  |  |  |  |  |
|  | **r** | **0.555** | **0.365** | **0.402** | **0.389** | **0.482** | **0.483** | **0.396** | **0.342** | **0.367** | **0.396** |  |  |  |  |  |  |
|  | **p** | **0.001** | **0.031** | **0.017** | **0.021** | **0.003** | **0.003** | **0.019** | **0.044** | **0.030** | **0.019** |  |  |  |  |  |  |
|  |  |  |  |  |  |  |  |  |  |  |  |  |  |  |  |  |  |
|  |  |  |  |  |  |  |  |  |  |  |  |  |  |  |  |  |  |
| **Sandstones** |  | **Ag** | **Al** | **As** | **Ba** | **Bi** | **Ca** | **Cd** | **Cr** | **Cu** | **Fe** | **Ga** | **K** | **Li** | **Mg** | **Mn** | **Na** |
|  | **r** | **0.354** | **0.382** | **0.424** | **0.474** | **0.417** | **0.573** | **0.438** | **0.538** | **0.368** | **0.601** | **0.389** | **0.644** | **0.347** | **0.516** | **0.375** | **0.417** |
|  | **p** | **0.037** | **0.024** | **0.011** | **0.004** | **0.013** | **0.000** | **0.001** | **0.001** | **0.030** | **0.000** | **0.021** | **0.000** | **0.041** | **0.001** | **0.027** | **0.013** |
|  |  |  |  |  |  |  |  |  |  |  |  |  |  |  |  |  |  |
| **Granites** |  | **Ag** | **Al** | **As** | **Bi** | **Ca** | **Ga** | **Ni** | **Pb** | **Sn** | **Th** | **W** | **Zn** |  |  |  |  |
|  | **r** | **-0.382** | **-0.350** | **-0.44** | **-0.473** | **-0.369** | **-0.440** | **-0.427** | **0.434** | **-0.634** | **-0.447** | **-0.595** | **-0.473** |  |  |  |  |
|  | **p** | **0.024** | **0.040** | **0.008** | **0.004** | **0.029** | **0.008** | **0.010** | **0.090** | **0.000** | **0.007** | **0.000** | **0.004** |  |  |  |  |
|  |  |  |  |  |  |  |  |  |  |  |  |  |  |  |  |  |  |
| **Phyllites** |  | **Mo** | **Sn** | **W** |  |  |  |  |  |  |  |  |  |  |  |  |  |
|  | **r** | **-0.338** | **0.383** | **0.42** |  |  |  |  |  |  |  |  |  |  |  |  |  |
|  | **p** | **0.047** | **0.023** | **0.012** |  |  |  |  |  |  |  |  |  |  |  |  |  |
|  |  |  |  |  |  |  |  |  |  |  |  |  |  |  |  |  |  |
| **Basalts** |  | **Bi** | **Mn** | **Rb** | **Sr** |  |  |  |  |  |  |  |  |  |  |  |  |
|  | **r** | **-0.344** | **-0.354** | **0.364** | **-0.364** |  |  |  |  |  |  |  |  |  |  |  |  |
|  | **p** | **0.043** | **0.037** | **0.032** | **0.032** |  |  |  |  |  |  |  |  |  |  |  |  |
|  |  |  |  |  |  |  |  |  |  |  |  |  |  |  |  |  |  |
|  |  |  |  |  |  |  |  |  |  |  |  |  |  |  |  |  |  |
|  |  |  |  |  |  |  |  |  |  |  |  |  |  |  |  |  |  |
|  |  |  |  |  |  |  |  |  |  |  |  |  |  |  |  |  |  |
|  |  |  |  |  |  |  |  |  |  |  |  |  |  |  |  |  |  |
|  |  |  |  |  |  |  |  |  |  |  |  |  |  |  |  |  |  |
