# Supplementary figures and images for: Which trace elements are accumulated in fronds of the Athyrium filix-femina fern? a study from the Czech Republic
Source: Environ Monit Assess. 2025 Jun 24;197(7):801. doi: 10.1007/s10661-025-14201-4 (PMC12187792; doi:10.1007/s10661-025-14201-4)

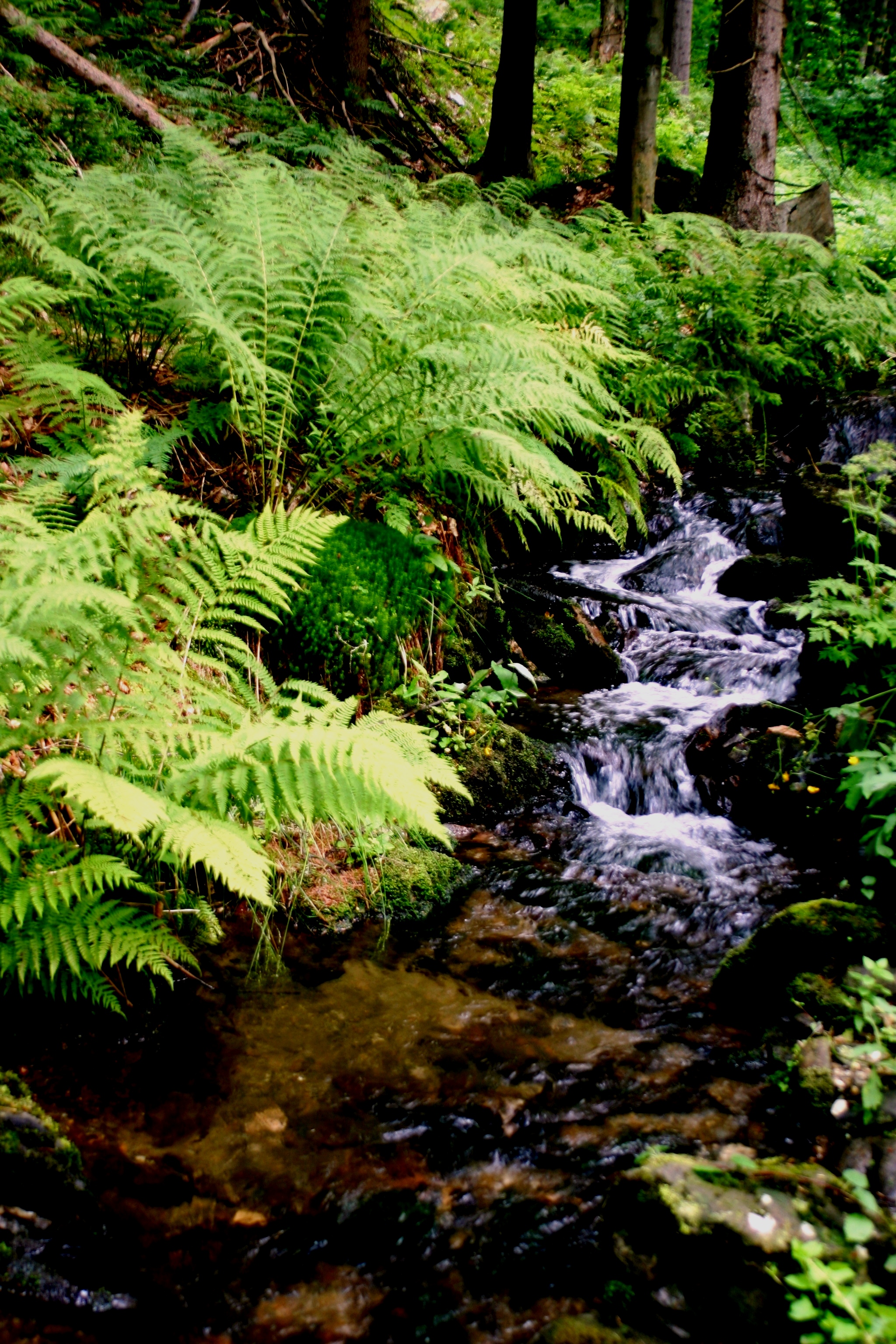

Supplement: Supplementary file 8 — Supplementary file8 (JPG 2720 KB) [file 10661_2025_14201_MOESM8_ESM.jpg]
